# Fluoxetine Administration in Juvenile Monkeys: Implications for Pharmacotherapy in Children

**DOI:** 10.3389/fped.2018.00021

**Published:** 2018-02-08

**Authors:** Mari S. Golub, Casey E. Hogrefe, Richard J. Sherwood, Christoph W. Turck

**Affiliations:** ^1^California National Primate Research Center, University of California, Davis, Davis, CA, United States; ^2^School of Medicine, University of Missouri, Columbia, MO, United States; ^3^Max Planck Institute of Psychiatry, Munich, Germany

**Keywords:** fluoxetine, children, monkeys, sleep, attention, cognition, metabolomics, monoamine oxidase A

## Abstract

Fluoxetine therapy has been approved for children with major depressive disorder and obsessive compulsive disorder for over 14 years and has expanded to other childhood behavior disorders. As use increases, more detail on fluoxetine effects during juvenile brain development can help maintain safe and effective use of this therapy. Here, a narrative review is provided of previously published findings from a large nonhuman primate project. Fluoxetine was administered to juvenile male rhesus monkeys for an extended period (2 years) prior to puberty. Compared to controls, treated monkeys showed sleep disruption, facilitated social interaction, greater impulsivity, and impaired sustained attention during treatment. No effects on growth were seen. Metabolomics assays characterized a distinctive response to fluoxetine and demonstrated individual differences that were related to the impulsivity measure. Fluoxetine interactions with monoamine oxidase A polymorphisms that influenced behavior and metabolomics markers were an important, previously unrecognized finding of our studies. After treatment was discontinued, some behavioral effects persisted, but short-term memory and cognitive flexibility testing did not show drug effects. This detailed experimental work can contribute to clinical research and continued safe and effective fluoxetine pharmacotherapy in children.

## Introduction

Fluoxetine is a selective serotonin reuptake inhibitor (SSRI) that was approved (as Prozac^®^) for use in adults for depression in 1987 and for use in children for depression (MDD) and obsessive compulsive disorder (OCD) in 2003. Expanding use of fluoxetine has spurred interest in filling the gap in basic and clinical research on effects of fluoxetine therapy specific to children. This review is based on a project undertaken to supplement information on the safety of fluoxetine for children by using a juvenile nonhuman primate model, the rhesus monkey. Macaque monkeys are prominent models for childhood because of the extended juvenile period between infancy and puberty when advanced cortical functions mature under the influence of complex social systems. The review integrates findings from eight published studies of different endpoint domains (1–8) with current issues in pediatric psychopharmacology.

## Juvenile Monkeys as a Model for Children

Species differences in drug metabolism can be an important barrier to translation. Studies in adult monkeys have demonstrated metabolic profiles of fluoxetine similar to those seen in humans (9–11), as did our pilot work in juvenile monkeys (1). Also, in that pilot research, fluoxetine’s biological action of blocking serotonin reuptake in brain was reflected in increased concentrations of serotonin in cerebral spinal fluid after long-term treatment (1).

Because simple weight-based extrapolation from humans to monkeys is not appropriate (11), preliminary pharmacokinetic studies in juvenile rhesus were used to select an oral dose of 2 mg/kg relevant to children (1). Plasma levels (fluoxetine + norfluoxetine) in the monkeys after 2 years of dosing were 273 ± 31 ng/mL (5), compared to 241 ± 91 ng/mL in children with MDD who showed a therapeutic response at the recommended dosage of 20 mg/day (12).

Life history provides a valuable parallel between monkeys and humans particularly as regards the prolonged period of development after infancy and before puberty known as childhood in humans. Monkeys in our project began dosing at 1 year of age (approximately equivalent to 4–6 years of age in children), were dosed for 2 years, and completed the postdosing follow-up at 4 years of age, just before puberty. An outline of the study schedule is provided in Table S1 in Supplementary Material.

Rhesus monkeys, like humans, display polymorphisms that result in greater or lesser expression of genes that regulate brain function and interact with environmental influences, including, prominently, monoamine oxidase A (MAOA), which metabolizes serotonin, and the serotonin transporter (SERT), which is responsible for reuptake of serotonin after release during neurotransmission (13). Genotyping for polymorphisms in these genes (MAOA-uVNTR and 5HTTLPR) was available in our monkey subjects. As it turned out, fluoxetine interactions with MAOA polymorphisms influenced several of the behavioral assessments, most prominently emotional response (Table S2 in Supplementary Material). Also, unexpectedly, 5HTTLPR polymorphisms interacted with fluoxetine in influencing growth.

## Limitations

Two major limitations of this project were the all-male cohort and the limited duration of postdosing follow-up.

## Experimental Findings

### Experimental Endpoints and Expectations

This project was purposely broad in scope and not designed to test specific hypotheses. However, the available literature did allow some expectations about the consequences of fluoxetine treatment in the juvenile monkeys. Most of the endpoints targeted for evaluation as potentially sensitive to fluoxetine were identified from clinical and experimental studies of SSRIs in human adults and children and from a small number of juvenile animal studies in the literature. This literature led to the following expectations for effects during dosing:

- Less growth in weight and height during treatment
- “Activation syndrome”: poor behavioral inhibition/hyperactivity
- Sleep disturbance, particularly nighttime awakening
- Facilitation of social interaction
- Deficit in sustained attention
- Altered emotional response
- Altered cortical synaptic spine density.

The complete schedule of evaluations is provided in Table S1 in Supplementary Material. Some of these expectations were supported in analysis of our nonhuman primate data and some were not.

### Less Growth in Weight and Height during Treatment

Effects on growth were a major finding in the 19-week clinical trial used to support FDA approval for fluoxetine use in children (14, 15), and the fluoxetine label recommends that “…height and weight should be monitored periodically in pediatric patients receiving fluoxetine.” A more recent 36-week clinical trial also reported decreased weight gain during fluoxetine treatment in children (16). In addition, the rat juvenile toxicity study conducted for FDA and described on the fluoxetine label (17) found growth retardation, but included postpubertal ages (weaning to adulthood).

The expectation of less growth was not supported in our study. There were no main effects of fluoxetine on growth in body weight, body length, long bone length, head circumference, arm circumference, or skinfold thickness measured at 4-month intervals (3).

However, nonhuman primate vs. human studies differed in several relevant respects including an all-male cohort and the lack of a depression diagnosis. Also, the human cohorts included adolescents whose rapid growth may have been more sensitive to the drug.

Unexpectedly, SERT 5HTTLPR genotype (LL vs. SL polymorphism) was found to influence growth rate in our nonhuman primate project (3). Growth was slower in the SL subjects (putative lower transcription) than the LL subjects (putative higher transcription) for body weight, body length, and long bone length during the second year of dosing. Interestingly, the 5HTTLPR effect on growth showed an interaction with fluoxetine. The slower growth in leg bones (femur and tibia) in the LL subjects was significant for the vehicle controls (*p* = 0.0006) but not for the fluoxetine-treated subjects (*p* = 0.41). This finding and related literature on serotonin and bone (18) suggests that 5HTTLPR may be an important consideration in conducting and replicating growth studies in juvenile monkeys and possibly children.

### “Activation Syndrome”

“Activation syndrome” is a term coined to describe side effects [or treatment-elicited adverse effects (TEAEs)] observed in response to SSRI therapy in children (19). The term includes symptoms with descriptors such as impulsivity, behavioral disinhibition, hyperactivity, jitteriness, and akathisia. “Activation syndrome” has been implicated in the early suicidality identified in SSRI-treated adolescents (20). As early as 1991, this pattern was reported in fluoxetine-treated children with depression (21) and has subsequently been observed in a number of studies in children treated for a variety of disorders. A single conceptual or biological basis has not been defined, but factor analysis of symptom reports indicates validity of the construct (22). In our study, we gathered information on this issue by measuring impulsivity with a reward delay task and hyperactivity with 48 h actimeter monitoring.

Our project found fluoxetine-induced impulsivity (2). In the reward delay task, the subject was required to withhold reaching for a food reward for 15 s, while it was gradually revealed from behind a screen. Fluoxetine-treated subjects responded sooner and had more immediate responses after 1 year of dosing. After 2 years of dosing, this group difference was no longer statistically significant (*p* = ~0.28). Notably, in children, activation side effects of SSRIs are more prominent in the youngest age groups (23).

Hyperactivity, as measured with actimeters, was not found in fluoxetine-treated monkeys (4). In children, an actimeter study of “activation syndrome” did not find that total daily activity was correlated with “activation syndrome” intensity as assessed by questionnaire (22).

### Sleep Disturbance, Particularly Nighttime Awakening

Sleep disturbance associated with SSRI administration was an early clinical observation later confirmed with EEG studies in adults, both patients and non-patients (24, 25). Measures included nighttime awakening, eye movement, restless legs, and, more recently, bruxism. In children, a similar finding was described during treatment with fluoxetine and other SSRIs in symptom reports (26, 27), as well as EEG/EOG monitoring (28).

Sleep disturbance was found in our non-human primate project (4). With our actimeter data, it was possible to use activity thresholds to identify the onset and duration of nighttime sleep, as well as disruption of sleep at night (awakenings), and daytime sleeping (naps). We measured sleep disruption with the Fragmentation Index, which combines number of nighttime awakenings and daytime naps. Fluoxetine led to more sleep disruption (greater Fragmentation Index) after both 1 and 2 years of dosing (4). An additional finding was that greater Fragmentation Index values in the fluoxetine group, as well as awakenings at night, were influenced by MAOA genotype (4). The fluoxetine effect was statistically significant in high-MAOA subjects but not low-MAOA subjects (Table S2 in Supplementary Material).

An actimeter study in children (22) found that “activation syndrome” was associated with more nighttime awakenings and less daytime activity. These authors suggested that sleep disturbance could serve as a marker for “activation syndrome.”

### Facilitation of Social Interaction

Fluoxetine has been used successfully in the treatment of social anxiety in both adults and children (29–31). An increased social interaction is also a well-recognized consequence of SSRI treatment of depression. While social facilitation may be a non-specific consequence of reduced anxiety and depression, there is also evidence of an independent drug effect from studies of social facilitation in adult non-patient populations given SSRIs (32–38).

**Figure 1:**
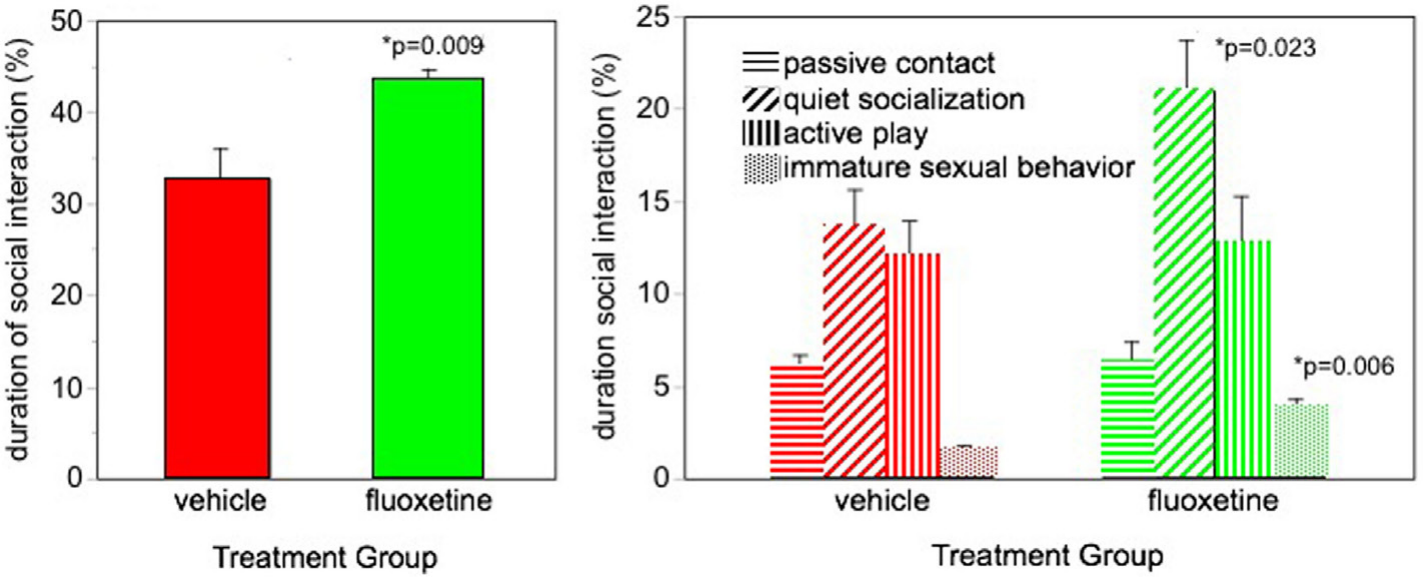
Social interaction. Percent of time spent in social interaction during three 30-min observation sessions over the 2-year dosing period. From Ref. (5). *N* = 16/group. *p*-values are for comparison of fluoxetine group with vehicle group.

Facilitated social interaction was seen in our fluoxetine-treated monkeys (5). Monkeys were housed in pairs and were observed interacting with their longtime cagemate. More social behavior was seen in the fluoxetine-treated subjects based on the sum of all social behavior (Figure 1). Also, the incidence of the most common type of social behavior, quiet socialization, was statistically greater in the fluoxetine-treated group. However, some specific types of behavior were differentially affected depending on the MAOA genotype: social invitations and initiations were greater in the high-MAOA-treated subject than the low-MAOA fluoxetine-treated subject, while grooming was enhanced in fluoxetine-treated dyads with two low-MAOA cagemates (Table S2 in Supplementary Material).

### Sustained Attention Impairment

Sustained attention is a cognitive domain that has been characterized in some depth for SSRI influences in adults (39–43). It has not been formally evaluated in children although poor attention is sometimes reported in TEAE questionnaires (23, 44–46).

**Figure 2:**
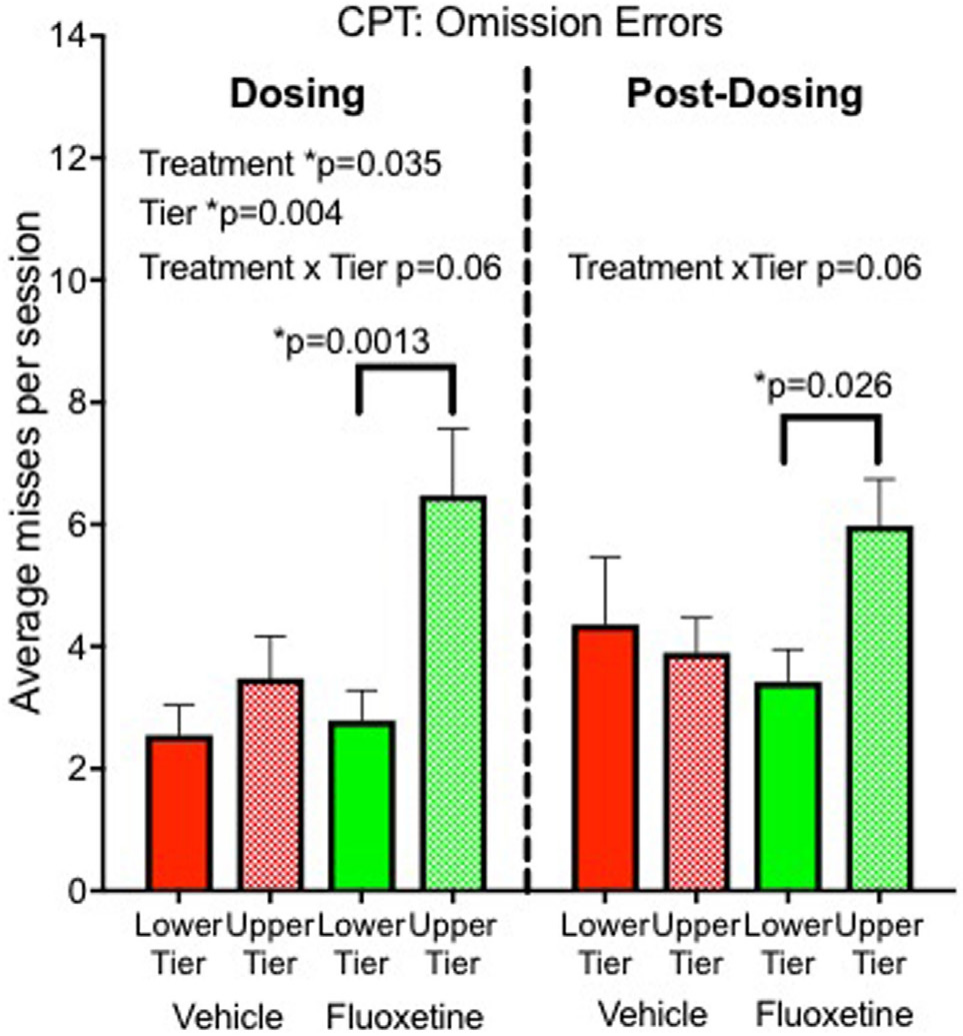
Sustained attention. Omission errors in the automated Continuous Performance Test administered 20 months after initiation of dosing (“Dosing”) and 6–10 months after discontinuation of dosing (“Post-Dosing”) (8).

Sustained attention was sensitive to fluoxetine in our nonhuman primate project (8). An automated Continuous Performance Test (CPT) was used to evaluate sustained attention at the end of the dosing period. Monkeys were rewarded with a sugar pellet for touching a white square when it appeared on the screen and punished with a delay for touching red or green squares. A total of 112 stimuli were presented during a 12-min session with different colored squares presented randomly. After 2 years of treatment, fluoxetine-treated monkeys showed more omission errors (failure to touch the correct stimulus) than vehicle controls, indicating a sustained attention deficit. The decrease in attention was measurable but not dramatic, resulting in 16% omission errors in the fluoxetine group compared to 11% in controls. Under the more challenging testing condition (upper tier caging), the omission rate was 23% in the fluoxetine-treated group (Figure 2).

Of interest, CPT commission errors (false alarms) were not influenced by fluoxetine, and there were no interactions with MAOA genotype.

### Altered Emotional Responsiveness

The mood and affect systems in the brain are a prime target of antidepressants. Empirical findings of therapeutic effects are often attributed to correction of dysfunction in these systems. However, with broad use of SSRIs in children, information is needed on mood and affect effects in the absence of mood disorders. By using imaging techniques in non-patient populations, antidepressants including fluoxetine have been shown to influence activity in brain circuits associated with emotional response (47, 48).

Our young nonhuman primate subjects were not selected for mood disorders. While one might anticipate that the emotional response would be altered by fluoxetine, the extent and direction of such an effect could not be predicted. To assess emotional response, we used picture-elicited emotion, a common tool in imaging studies of brain circuits underlying emotion, including adult studies with fluoxetine (49, 50) and basic research in children (51). Monkeys were shown pictures with neutral, positive affective, and negative affective content. Vocalizations, facial expressions, and behaviors associated with emotional response were scored from videotape (6).

**Figure 3:**
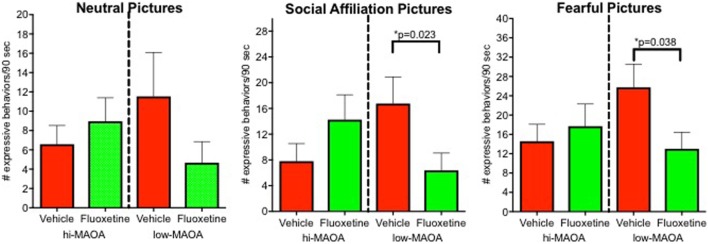
Emotional response. Emotional responses to pictures varying in affective content (neutral, social, and fearful). Responses were from an observational ethogram including vocalizations, facial expressions, and actions. From Ref. (6).

Emotional response was the major domain from our project that demonstrated a fluoxetine effects primarily associated with one MAOA genotype (6) (Figure 3; Table S2 in Supplementary Material). Low-MAOA subjects treated with fluoxetine were less emotionally reactive than their vehicle-treated counterparts. This result may be relevant to the inconsistent therapeutic efficacy of fluoxetine in childhood MDD, but also to fluoxetine use in conditions other than depression.

### Postdosing Persistence of Behavioral Effects

After discontinuation of dosing and a washout period, most of the assessments were repeated to look for persistence of effects. Persistent effects might stimulate further work on the more general question of whether exposure to this powerful serotonin system agent can alter the trajectory of brain development (52). In addition to the tests administered during dosing, two further tests of cognitive function that were still immature during the dosing period were added: recognition memory and cognitive flexibility. A simple comparison of drug effects during and after dosing was complicated by the rapid maturation of behavior that provided different background for detecting drug effects. However, some general conclusions on persistence can be offered at different levels of confidence.

- Impulsivity: marginal statistical significance (*p* = 0.055) for persistence (unpublished data).
- Sleep disturbance: fluoxetine effect on nighttime awakenings persisted (4).
- Sustained attention: persisted in subjects tested in the top tier (Figure 2) (8).
- Social interaction: fluoxetine effect on sum of all social interactions did not persist (unpublished data).
- Emotional response: emotional response was decreased in the low-MAOA genotype monkeys, as was the case during dosing, but the statistical test was not significant (*p* = 0.18) (6).

The two additional cognitive domains were evaluated only postdosing (short-term memory, cognitive flexibility) (8). The previous fluoxetine dosing did not impair memory or cognitive flexibility in these tests. However, subject engagement with testing was impaired in terms of trial initiation and completion (8). Multivariate analysis suggested that this was related to the persisting sustained attention deficit (8).

## Cortical Dendritic Synaptic Spine Density

In a different study in rhesus (53), enhanced expression of the SERT was found in cortex 1.5 years after discontinuation of a 1-year fluoxetine exposure during juvenile brain development. This finding demonstrates the possibility of long-term changes in brain after developmental fluoxetine treatment.

After conclusion of our project, we were able to complete an assessment of synaptic spine density in the monkey brains (8). Synaptic pruning is a hallmark of juvenile brain development in children, which is well documented in monkeys (54), and has been shown to be influenced by fluoxetine in rodent models (55–57). In cortex (dorsolateral prefrontal cortex), there was a pattern of lower density in fluoxetine-treated monkeys. However, this pattern could not be confirmed statistically in our sample (*N* = 8–9/group).

## Metabolomic Biomarkers of Response

Precision medicine seeks to use biomarkers to optimize therapeutic responses and minimize adverse side effects. There are several reasons why biomarkers that predict response to fluoxetine in children would be valuable. On the therapeutic side, looking specifically at MDD and OCD, the response rate to fluoxetine in children is similar to adults, about 57% of patient population compared to 33% placebo in a recent clinical trial (16). No approach to predicting responders vs. non-responders is currently available although genetics are beginning to be explored (58). In children, as in adults, the therapeutic response lags in time from the onset of treatment requiring prolonged dosing as the only way to determine therapeutic efficacy. On the adverse effects side, the postapproval emergence of reports of suicidality in adolescents treated with SSRIs emphasizes the value of predicting individual adverse side effects.

Metabolomics is one approach to providing biomarkers that predict treatment response and treatment-related adverse effects (59). In rodents and adult humans, metabolomics is already actively used to look for biomarkers of response to antidepressants (60–62). In our nonhuman primates, we looked for metabolomic biomarkers of fluoxetine response after 1 year of dosing.

Peripheral metabolite profiling was conducted using plasma, CSF, and fibroblasts from our juvenile monkeys (2, 7). To understand whether biomarkers of fluoxetine response predicted relevant behavioral effects, we correlated metabolomic biomarkers with impulsivity, a behavioral test affected by fluoxetine at 1 year of age. Finally, to further define individual response to fluoxetine, we included MAOA genotype in the analyses.

**Figure 4:**
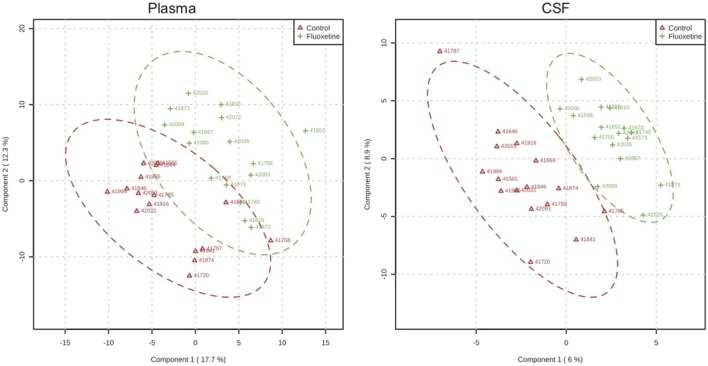
Metabolomic profiles. Partial least square analysis of metabolite profiles in plasma and CSF after 1 year of dosing demonstrating greater overlap for plasma than CSF samples. From Ref. (2).

In targeted analysis of plasma and CSF samples, partial least squares discriminant analysis demonstrated separation of metabolite profiles from control and fluoxetine-treated groups (2) (Figure 4). The separation was greater for CSF than for plasma. Two pathways emerged that distinguished fluoxetine and vehicle-treated animals and also contained metabolites that were associated with impulsivity: the *Alanine, Aspartate, Glutamate* pathway, and the *Nicotinate, Nicotinamide* pathway (2). This latter pathway was also influenced by MAOA genotype. It is directly linked to the metabolism of tryptophan, the amino acid precursor of serotonin, and to the *Alanine, Aspartate, Glutamate* pathway.

Metabolites in serum and CSF are subject to time-dependent “noise” from the environment. Fibroblasts are an easily obtainable biopsy material that has been recently added to biomarker studies of psychopathology (63, 64) to identify more stable biomarker profiles. When we looked at fibroblasts, the *Purine, Pyrimidine* and *Histidine* metabolic pathways were influenced by drug (7). Somewhat surprisingly, a strong biomarker for impulsivity was identified in the *Pyrimidine* pathway of the fibroblasts. Our project reinforces recent research showing a possible involvement of *Purine* and *Pyrimidine* pathways in adult mood disorders and their response to antidepressants (62, 65–67). *Purine* pathway metabolites previously implicated as biomarkers of therapeutic response to fluoxetine in depressed adult patients (65) may also be relevant to children.

## New Findings of Potential Value for Clinical Translation

In addition to confirming some previously described effects of fluoxetine in a developmental context, a few entirely new findings were generated in the project.

(1) Interaction of fluoxetine with MAOA genotype. Together with research on the interactions of developmental environment with MAOA polymorphism, these findings support the important role of these common gene variants in determining later behavior.
(2) Bone growth effects in conjunction with 5HTTLPR polymorphism. The broad utilization of serotonin in tissues outside the nervous system is well known. A literature is also developing for serotonin systems in the bone. Implications of developmental SSRI use for tissues other than brain may need attention in children.
(3) Biomarkers. Continued research on minimally invasive biological sampling may yield potentially useful biomarkers for predicting response to fluoxetine in children.

## Author Contributions

MG was the PI for the NIH grant that supported this work. She also wrote the manuscript and is responsible for recruiting the collaborating authors. CH was a principle technician, established protocols, scheduled and supervised all work, and reviewed the manuscript. RS contributed to protocols and data analysis of skeletal radiographs. CT collaborated as a coinvestigator, conducted metabolomic assays, and supervised data analysis and publication of metabolomic data.

## Conflict of Interest Statement

The authors report no biomedical financial interest and no potential conflicts of interest.

## References

[1] Golub MS Hogrefe CE. Fluoxetine: juvenile pharmacokinetics in a nonhuman primate model. Psychopharmacology (Berl) (2014) 231(20):4041–7. 10.1007/s00213-014-3537-y 24700388 PMC4176515

[2] He Y Hogrefe CE Grapov D Palazoglu M Fiehn O Turck CW Identifying individual differences of fluoxetine response in juvenile rhesus monkeys by metabolite profiling. Transl Psychiatry (2014) 4:e478. 10.1038/tp.2014.116 25369145 PMC4259988

[3] Golub MS Bulleri AM Hogrefe CE Sherwood RJ. Bone growth in juvenile rhesus monkeys is influenced by 5HTTLPR polymorphisms and interactions between 5HTTLPR polymorphisms and fluoxetine. Bone (2015) 79:162–9. 10.1016/j.bone.2015.05.042 26067181 PMC4511468

[4] Golub MS Hogrefe CE. Sleep disturbance as detected by actigraphy in pre-pubertal juvenile monkeys receiving therapeutic doses of fluoxetine. Neurotoxicol Teratol (2016) 55:1–7. 10.1016/j.ntt.2016.02.006 26956991 PMC4884518

[5] Golub MS Hogrefe CE Bulleri AM Peer social interaction is facilitated in juvenile rhesus monkeys treated with fluoxetine. Neuropharmacology (2016) 105:553–60. 10.1016/j.neuropharm.2016.02.025 26905291 PMC4873333

[6] Golub MS Hogrefe CE Bulleri AM Regulation of emotional response in juvenile monkeys treated with fluoxetine: MAOA interactions. Eur Neuropsychopharmacol (2016) 26(12):1920–9. 10.1016/j.euroneuro.2016.10.010 27852517 PMC5154301

[7] Su SY Hogrefe-Phi CE Asara JM Turck CW Golub MS. Peripheral fibroblast metabolic pathway alterations in juvenile rhesus monkeys undergoing long-term fluoxetine administration. Eur Neuropsychopharmacol (2016) 26(7):1110–8. 10.1016/j.euroneuro.2016.03.017 27084303 PMC5590669

[8] Golub MS Hackett EP Hogrefe CE Leranth C Elsworth J Roth R. Cognitive performance of juvenile monkeys after chronic fluoxetine treatment. Dev Cogn Neurosci (2017) 26:52–61. 10.1016/j.dcn.2017.04.008 28521247 PMC5557667

[9] Fontenot MB Padgett EE III Dupuy AM Lynch CR De Petrillo PB Higley JD. The effects of fluoxetine and buspirone on self-injurious and stereotypic behavior in adult male rhesus macaques. Comp Med (2005) 55(1):67–74. 15766212

[10] Fontenot MB Musso MW McFatter RM Anderson GM. Dose-finding study of fluoxetine and venlafaxine for the treatment of self-injurious and stereotypic behavior in rhesus macaques (*Macaca mulatta*). J Am Assoc Lab Anim Sci (2009) 48(2):176–84. 19383215 PMC2679666

[11] Sawyer EK Howell LL. Pharmacokinetics of fluoxetine in rhesus macaques following multiple routes of administration. Pharmacology (2011) 88(1–2):44–9. 10.1159/000329417 21757974 PMC3595567

[12] Koelch M Pfalzer AK Kliegl K Rothenhofer S Ludolph AG Fegert JM Therapeutic drug monitoring of children and adolescents treated with fluoxetine. Pharmacopsychiatry (2012) 45(2):72–6. 10.1055/s-0031-1291294 22086744

[13] Duncan LE Pollastri AR Smoller JW Mind the gap: why many geneticists and psychological scientists have discrepant views about gene-environment interaction (GxE) research. Am Psychol (2014) 69(3):249–68. 10.1037/a0036320 24750075 PMC7446184

[14] Mosholder A Medical Review: Center for Drug Evaluation and Research: Application Number 18-936/SE5-064. U S Food and Drug Administration (2002).

[15] Nilsson M Joliat MJ Miner CM Brown EB Heiligenstein JH. Safety of subchronic treatment with fluoxetine for major depressive disorder in children and adolescents. J Child Adolesc Psychopharmacol (2004) 14(3):412–7. 10.1089/cap.2004.14.412 15650497

[16] Emslie GJ Wells TG Prakash A Zhang Q Pangallo BA Bangs ME Acute and longer-term safety results from a pooled analysis of duloxetine studies for the treatment of children and adolescents with major depressive disorder. J Child Adolesc Psychopharmacol (2015) 25(4):293–305. 10.1089/cap.2014.0076 25978741

[17] US Food and Drug Administration. Prozac (Fluoxetine Hydrochloride) Capsules Label. (2017). Available from: https://www.accessdata.fda.gov/drugsatfda_docs/label/2011/018936s091lbl.pdf

[18] Calarge CA Ivins SD Motyl KJ Shibli-Rahhal AA Bliziotes MM Schlechte JA. Possible mechanisms for the skeletal effects of antipsychotics in children and adolescents. Ther Adv Psychopharmacol (2013) 3(5):278–93. 10.1177/2045125313487548 24167704 PMC3805387

[19] Amitai M Chen A Weizman A Apter A SSRI-induced activation syndrome in children and adolescents-what is next. Curr Treat Options Psychiatry (2015) 2(1):28–37. 10.1007/s40501-015-0034-9

[20] Hammad TA Laughren T Racoosin J. Suicidality in pediatric patients treated with antidepressant drugs. Arch Gen Psychiatry (2006) 63(3):332–9. 10.1001/archpsyc.63.3.332 16520440

[21] Riddle MA King RA Hardin MT Scahill L Ort SI Chappell P Behavioral side effects of fluoxetine in children and adolescents. J Child Adolesc Psychopharmacol (1990) 1(3):193–8. 10.1089/cap.1990.1.193

[22] Bussing R Reid AM McNamara JP Meyer JM Guzick AG Mason DM A pilot study of actigraphy as an objective measure of SSRI activation symptoms: results from a randomized placebo controlled psychopharmacological treatment study. Psychiatry Res (2015) 225(3):440–5. 10.1016/j.psychres.2014.11.070 25535011 PMC4428142

[23] Safer DJ. Raising the minimum effective dose of serotonin reuptake inhibitor antidepressants: adverse drug events. J Clin Psychopharmacol (2016) 36(5):483–91. 10.1097/JCP.0000000000000564 27518478

[24] Armitage R Trivedi M Rush AJ. Fluoxetine and oculomotor activity during sleep in depressed patients. Neuropsychopharmacology (1995) 12(2):159–65. 10.1016/0893-133x(94)00075-b 7779244

[25] Armitage R Yonkers K Cole D Rush AJ A multicenter, double-blind comparison of the effects of nefazodone and fluoxetine on sleep architecture and quality of sleep in depressed outpatients. J Clin Psychopharmacol (1997) 17(3):161–8. 10.1097/00004714-199706000-00004 9169959

[26] Jain U Birmaher B Garcia M Al-Shabbout M Ryan N. Fluoxetine in children and adolescents with mood disorders: a chart review of efficacy and adverse effects. J Child Adolesc Psychopharmacol (1992) 2(4):259–65. 10.1089/cap.1992.2.259 19630607

[27] Wilens TE Biederman J Kwon A Chase R Greenberg L Mick E A systematic chart review of the nature of psychiatric adverse events in children and adolescents treated with selective serotonin reuptake inhibitors. J Child Adolesc Psychopharmacol (2003) 13(2):143–52. 10.1089/104454603322163862 12886909

[28] Armitage R Emslie G Rintelmann J The effect of fluoxetine on sleep EEG in childhood depression: a preliminary report. Neuropsychopharmacology (1997) 17(4):241–5. 10.1016/S0893-133X(97)00048-1 9326748

[29] Birmaher B Axelson DA Monk K Kalas C Clark DB Ehmann M Fluoxetine for the treatment of childhood anxiety disorders. J Am Acad Child Adolesc Psychiatry (2003) 42(4):415–23. 10.1097/01.CHI.0000037049.04952.9F 12649628

[30] Beidel DC Turner SM Sallee FR Ammerman RT Crosby LA Pathak S. SET-C versus fluoxetine in the treatment of childhood social phobia. J Am Acad Child Adolesc Psychiatry (2007) 46(12):1622–32. 10.1097/chi.0b013e318154bb57 18030084

[31] Hedges DW Brown BL Shwalb DA Godfrey K Larcher AM. The efficacy of selective serotonin reuptake inhibitors in adult social anxiety disorder: a meta-analysis of double-blind, placebo-controlled trials. J Psychopharmacol (2007) 21(1):102–11. 10.1177/0269881106065102 16714326

[32] Knutson B Wolkowitz OM Cole SW Chan T Moore EA Johnson RC Selective alteration of personality and social behavior by serotonergic intervention. Am J Psychiatry (1998) 155(3):373–9. 10.1176/ajp.155.3.373 9501748

[33] Tse WS Bond AJ. Serotonergic involvement in the psychosocial dimension of personality. J Psychopharmacol (2001) 15(3):195–8. 10.1177/026988110101500313 11565628

[34] Tse WS Bond AJ Difference in serotonergic and noradrenergic regulation of human social behaviours. Psychopharmacology (Berl) (2002) 159(2):216–21. 10.1007/s00213-001-0926-9 11862352

[35] Tse WS Bond AJ Serotonergic intervention affects both social dominance and affiliative behaviour. Psychopharmacology (Berl) (2002) 161(3):324–30. 10.1007/s00213-002-1049-7 12021836

[36] Tse WS Bond AJ. Reboxetine promotes social bonding in healthy volunteers. J Psychopharmacol (2003) 17(2):189–95. 10.1177/0269881103017002007 12870566

[37] Tse WS Bond AJ. Noradrenaline might enhance assertive human social behaviours: an investigation in a flatmate relationship. Pharmacopsychiatry (2006) 39(5):175–9. 10.1055/s-2006-948328 16944408

[38] Tse WS Chow H Wing YK Bond AJ. Using a partner’s facial emotion to elucidate social dominance motivation induced by an SSRI. Eur Neuropsychopharmacol (2014) 24(10):1641–9. 10.1016/j.euroneuro.2014.07.011 25169642

[39] Ramaekers JG Muntjewerff ND O’Hanlon JF. A comparative study of acute and subchronic effects of dothiepin, fluoxetine and placebo on psychomotor and actual driving performance. Br J Clin Pharmacol (1995) 39(4):397–404. 10.1111/j.1365-2125.1995.tb04468.x 7640146 PMC1365127

[40] O’Hanlon JF Robbe HW Vermeeren A van Leeuwen C Danjou PE. Venlafaxine’s effects on healthy volunteers’ driving, psychomotor, and vigilance performance during 15-day fixed and incremental dosing regimens. J Clin Psychopharmacol (1998) 18(3):212–21. 10.1097/00004714-199806000-00006 9617980

[41] Schmitt JA Ramaekers JG Kruizinga MJ van Boxtel MP Vuurman EF Riedel WJ. Additional dopamine reuptake inhibition attenuates vigilance impairment induced by serotonin reuptake inhibition in man. J Psychopharmacol (2002) 16(3):207–14. 10.1177/026988110201600303 12236626

[42] Riedel WJ Eikmans K Heldens A Schmitt JA. Specific serotonergic reuptake inhibition impairs vigilance performance acutely and after subchronic treatment. J Psychopharmacol (2005) 19(1):12–20. 10.1177/0269881105048887 15671124

[43] Wingen M Kuypers KP van de Ven V Formisano E Ramaekers JG. Sustained attention and serotonin: a pharmaco-fMRI study. Hum Psychopharmacol (2008) 23(3):221–30. 10.1002/hup.923 18257001

[44] Safer DJ Zito JM. Treatment-emergent adverse events from selective serotonin reuptake inhibitors by age group: children versus adolescents. J Child Adolesc Psychopharmacol (2006) 16(1–2):159–69. 10.1089/cap.2006.16.159 16553536

[45] Safer DJ. Age-grouped differences in adverse drug events from psychotropic medication. J Child Adolesc Psychopharmacol (2011) 21(4):299–309. 10.1089/cap.2010.0152 21851188

[46] Lee CS Williamson LR Martin SE DeMarco M Majczak M Martini J Adverse events in very young children prescribed psychotropic medications: preliminary findings from an acute clinical sample. J Child Adolesc Psychopharmacol (2015) 25(6):509–13. 10.1089/cap.2015.0034 26262905

[47] Norbury R Taylor MJ Selvaraj S Murphy SE Harmer CJ Cowen PJ. Short-term antidepressant treatment modulates amygdala response to happy faces. Psychopharmacology (Berl) (2009) 206(2):197–204. 10.1007/s00213-009-1597-1 19585106

[48] Pringle A Harmer CJ. The effects of drugs on human models of emotional processing: an account of antidepressant drug treatment. Dialogues Clin Neurosci (2015) 17(4):477–87. 26869848 10.31887/DCNS.2015.17.4/apringle PMC4734885

[49] Tao R Calley CS Hart J Mayes TL Nakonezny PA Lu H Brain activity in adolescent major depressive disorder before and after fluoxetine treatment. Am J Psychiatry (2012) 169(4):381–8. 10.1176/appi.ajp.2011.11040615 22267183 PMC4225078

[50] Rizvi SJ Salomons TV Konarski JZ Downar J Giacobbe P McIntyre RS Neural response to emotional stimuli associated with successful antidepressant treatment and behavioral activation. J Affect Disord (2013) 151(2):573–81. 10.1016/j.jad.2013.06.050 23948629

[51] Perlman SB Hein TC Stepp SD LAMS Consortium. Emotional reactivity and its impact on neural circuitry for attention-emotion interaction in childhood and adolescence. Dev Cogn Neurosci (2014) 8:100–9. 10.1016/j.dcn.2013.08.005 24055416 PMC3949237

[52] Christian RB Gaynes BN Saavedra LM Sheitman B Wines R Jonas DE Use of antipsychotic medications in pediatric and young adult populations: future research needs. J Psychiatr Pract (2015) 21(1):26–36. 10.1097/01.pra.0000460619.10429.4c 25603449

[53] Shrestha SS Nelson EE Liow JS Gladding R Lyoo CH Noble PL Fluoxetine administered to juvenile monkeys: effects on the serotonin transporter and behavior. Am J Psychiatry (2014) 171(3):323–31. 10.1176/appi.ajp.2013.13020183 24480874 PMC4181537

[54] Anderson SA Classey JD Conde F Lund JS Lewis DA. Synchronous development of pyramidal neuron dendritic spines and parvalbumin-immunoreactive chandelier neuron axon terminals in layer III of monkey prefrontal cortex. Neuroscience (1995) 67(1):7–22. 10.1016/0306-4522(95)00051-J 7477911

[55] Norrholm SD Ouimet CC. Chronic fluoxetine administration to juvenile rats prevents age-associated dendritic spine proliferation in hippocampus. Brain Res (2000) 883(2):205–15. 10.1016/S0006-8993(00)02909-7 11074049

[56] Hajszan T MacLusky NJ Leranth C. Short-term treatment with the antidepressant fluoxetine triggers pyramidal dendritic spine synapse formation in rat hippocampus. Eur J Neurosci (2005) 21(5):1299–303. 10.1111/j.1460-9568.2005.03968.x 15813939

[57] Zheng J Xu DF Li K Wang HT Shen PC Lin M Neonatal exposure to fluoxetine and fluvoxamine alteres spine density in mouse hippocampal CA1 pyramidal neurons. Int J Clin Exp Pathol (2011) 4(2):162–8. 21326811 PMC3037202

[58] Gasso P Rodriguez N Mas S Pagerols M Blazquez A Plana MT Effect of CYP2D6, CYP2C9 and ABCB1 genotypes on fluoxetine plasma concentrations and clinical improvement in children and adolescent patients. Pharmacogenomics J 14(5):457–62. (2014). 10.1038/tpj.2014.12 24663076

[59] Beger RD Dunn W Schmidt MA Gross SS Kirwan JA Cascante M Metabolomics enables precision medicine: “a white paper, community perspective”. Metabolomics (2016) 12(10):149 10.1007/s11306-016-1094-6 27642271 PMC5009152

[60] Webhofer C Gormanns P Tolstikov V Zieglgansberger W Sillaber I Holsboer F Metabolite profiling of antidepressant drug action reveals novel drug targets beyond monoamine elevation. Transl Psychiatry (2011) 1:e58. 10.1038/tp.2011.56 22832350 PMC3309495

[61] Weckmann K Labermaier C Asara JM Muller MB Turck CW. Time-dependent metabolomic profiling of ketamine drug action reveals hippocampal pathway alterations and biomarker candidates. Transl Psychiatry (2014) 4:e481. 10.1038/tp.2014.119 25386958 PMC4259990

[62] Park DI Dournes C Sillaber I Uhr M Asara JM Gassen NC Purine and pyrimidine metabolism: convergent evidence on chronic antidepressant treatment response in mice and humans. Sci Rep (2016) 6:35317. 10.1038/srep35317 27731396 PMC5059694

[63] Gasso P Mas S Molina O Lafuente A Bernardo M Parellada E Increased susceptibility to apoptosis in cultured fibroblasts from antipsychotic-naive first-episode schizophrenia patients. J Psychiatr Res (2014) 48(1):94–101. 10.1016/j.jpsychires.2013.09.017 24128664

[64] Batalla A Bargallo N Gasso P Molina O Pareto D Mas S Apoptotic markers in cultured fibroblasts correlate with brain metabolites and regional brain volume in antipsychotic-naive first-episode schizophrenia and healthy controls. Transl Psychiatry (2015) 5:e626. 10.1038/tp.2015.122 26305477 PMC4564572

[65] Renshaw PF Parow AM Hirashima F Ke Y Moore CM Frederick Bde B Multinuclear magnetic resonance spectroscopy studies of brain purines in major depression. Am J Psychiatry (2001) 158(12):2048–55. 10.1176/appi.ajp.158.12.2048 11729024

[66] Burnstock G Krugel U Abbracchio MP Illes P. Purinergic signalling: from normal behaviour to pathological brain function. Prog Neurobiol (2011) 95(2):229–74. 10.1016/j.pneurobio.2011.08.006 21907261

[67] Lindberg D Shan D Ayers-Ringler J Oliveros A Benitez J Prieto M Purinergic signaling and energy homeostasis in psychiatric disorders. Curr Mol Med (2015) 15(3):275–95. 10.2174/1566524015666150330163724 25950756 PMC5036858

