# Supplementary material for: Fluoxetine Administration in Juvenile Monkeys: Implications for Pharmacotherapy in Children
Source: Front Pediatr. 2018 Feb 8;6:21. doi: 10.3389/fped.2018.00021 (PMC5809484; doi:10.3389/fped.2018.00021)
Supplement: Supplementary file 1 [file table_1.docx]

Table S1. Complete testing and evaluation schedule. Fluoxetine dosing was conducted from 1-3 years of age. Time of evaluations is shown as months from initiation of dosing (1 year of age). CSF=cerebrospinal fluid; PET=positron emission tomography; MRI=magnetic resonance imaging; CPT=Continuous Performance Task;DNMS=Delayed non-matching to sample; IDED=Intra-dimensional/Extra-dimensional shift

| Test/Evaluation | During Dosing | Post-Dosing |
| --- | --- | --- |
| Morphometric exams | Bimonthly, 0-24 months | Bimonthly, 26-36 months |
| Long bone radiographs | 0, 12, 24 months | 28, 32, 36 months |
| Puberty assessment |  | Bimonthly, 26-36 months |
| Metabolomics samples (plasma, CSF, fibroblasts) | 12 months | 36 months (no fibroblasts) |
| PET and MRI | -- | 37-38 months |
| Social Dyad | 6, 12, 20 months | 26, 36 months |
| Impulsivity | 12, 24 months | 36 months |
| Picture Elicited Emotion | 12, 24 months | 36 months |
| Activity Monitoring | 12, 24 months | 36 months |
| CANTAB Cognitive Testing |  |  |
| CPT | 20 months | 30-34 months |
| DNMS, IDED | -- | 29-31 months |
| Vet exam, necropsy and brain collection | -- | 38-39 months |
