# Supplementary material for: Fluoxetine Administration in Juvenile Monkeys: Implications for Pharmacotherapy in Children
Source: Front Pediatr. 2018 Feb 8;6:21. doi: 10.3389/fped.2018.00021 (PMC5809484; doi:10.3389/fped.2018.00021)
Supplement: Supplementary file 2 [file table_2.docx]

Table S2. Fluoxetine interactions with MAOA genotype

| **Domain** | **Fluoxetine*MAOA genotype interaction** |
| --- | --- |
|  |  |
| Activity & Sleep | Fluoxetine increase in sleep fragmentation was greater in the high-MAOA subjects |
|  |  |
| Social Interaction | In the fluoxetine group, more behavior invitations and initiations were seen in the high-MAOA subjects than the low MAOA subjects. |
|  |  |
| Emotional response to pictures | Fluoxetine decreased emotional response in the low-MAOA group. |
| Short term memory test | Fluoxetine decreased the number of trial initiations in the high-MAOA group. |
|  |  |
